# Enhancing Brain–Computer Interfaces through Kriging-Based Fusion of Sparse Regression Partial Differential Equations to Counter Injection Molding View of Node Displacement Effects

**DOI:** 10.3390/polym16172507

**Published:** 2024-09-03

**Authors:** Hanjui Chang, Yue Sun, Shuzhou Lu, Yuntao Lan

**Affiliations:** 1 Department of Mechanical Engineering, College of Engineering, Shantou University, Shantou 515063, China; 22ysun@stu.edu.cn (Y.S.); 21szlu@stu.edu.cn (S.L.); 13433681239@163.com (Y.L.); 2 Intelligent Manufacturing Key Laboratory of Ministry of Education, Shantou University, Shantou 515063, China

**Keywords:** brain–computer interface (BCI), in-mold electronics (IME), Kriging, node displacement, PDEs

## Abstract

**Highlights:**

- IME technology is combined with conductive PET and LSR polymer materials to preform Utah arrays for BCI.
- The effects of three key factors on the nodal displacement were analyzed using the Kriging model.
- The heat transfer during injection molding was further analyzed by PDEs in order to determine the parameters more accurately.
- The allowable variation range of the wire diameter is obtained by the relation between the wire diameter and the current.

**Abstract:**

Injection molding is an efficient and precise manufacturing technology that is widely used in the production of plastic products. In recent years, injection molding technology has made significant progress, especially with the combination of in-mold electronics (IME) technology, which makes it possible to embed electronic components directly into the surface of a product. IME technology improves the integration and performance of a product by embedding conductive materials and functional components in the mold. Brain–computer interfaces (BCIs) are a rapidly growing field of research that aims to capture, analyze, and feedback brain signals by directly connecting the brain to external devices. The Utah array, a high-density microelectrode array, has been widely used for the recording and transmission of brain signals. However, the traditional fabrication method of the Utah array suffers from high cost and low integration, which limits its promotion in practical applications. The lines that receive EEG signals are one of the key parts of a brain–computer interface system. The optimization of injection molding parameters is particularly important in order to effectively embed these lines into thin films and to ensure the precise displacement of the line nodes and the stability of signal transmission during the injection molding process. In this study, a method based on the Kriging prediction model and sparse regression partial differential equations (PDEs) is proposed to optimize the key parameters in the injection molding process. This method can effectively predict and control the displacement of nodes in the film, ensure the stability and reliability of the line during the injection process, and improve the accuracy of EEG signal transmission and system performance. The optimal injection parameters were finally obtained: a holding pressure of 525 MPa, a holding time of 50 s, and a melting temperature of 285 °C. Under this condition, the average node displacement of UA was reduced from the initial 0.19 mm to 0.89 µm, with an optimization rate of 95.32%.

## 1. Introduction

Brain–computer interface (BCI) technology is a cutting-edge interdisciplinary area combining neuroscience, computer science, and engineering to establish a direct connection between the human brain and external devices. With the rapid development of neuroscience and computer science, BCI technology provides new ways for people to explore direct interaction with computers or other intelligent devices [1].

In the medical field, embedded brain–computer interface technology is of great significance. For patients who have lost their motor abilities due to brain injury, amputation, or neurological disorders, BCI technology offers a path of hope [2]. By recording and analyzing brain activity, BCI can convert brain signals into commands that control assistive devices such as prosthetic limbs or exoskeletons [3]. These applications can help patients regain some motor function and improve quality of life and autonomy.

In neuroscience research, embedded brain–computer interface technology shows great potential for development. By directly recording brain activities, scientists can gain a deeper understanding of human brain functions and cognitive processes [4]. BCI technology helps neuroscientists decipher the brain’s information-processing mechanisms, which can lead to in-depth studies of complex neural behaviors such as memory, learning, and decision-making [5,6,7].

In brain–computer interfaces, a microelectrode array is a set of tens to hundreds of micrometer-sized electrodes that are implanted or attached directly to the surface of the cerebral cortex to capture electrical signals from the brain by recording neuronal activity. The rigidity of the electrodes and the flexibility of the mesh substrate allow them to penetrate tissues and fit tightly to the brain surface to effectively capture neural signals [8]. However, long-term electrode stability and variability are key challenges in BCI research and clinical applications [9].

A Utah array consists of a set of tiny electrodes that are precisely implanted in specific areas of the cerebral cortex by microsurgery, enabling the real-time recording and monitoring of neuronal electrical activity and capturing subtle changes in the brain [10]. In addition, microelectrode insertion sites can modulate inflammation, promote neuroprotection, and improve performance over time [11].

Brain–computer interface implantation is a complex and challenging task involving the implantation of electrodes or other sensors into the brain to capture neural signals and enable interaction with external devices [12]. In-mold electronics (IME), an emerging fabrication technology, meets the needs of Utah arrays by combining printed electronic components with thin-film insertion molding to fabricate highly integrated, small-sized electronic devices [13,14].

The occurrence of joint displacement is closely related to the flow behavior in the injection molding process, the pressure distribution in the pressure holding stage, the shrinkage in the cooling process and the mold design. In the process of filling the mold cavity with molten plastic, factors such as the flow speed of the plastic, shear stress, and pressure distribution will exert forces on the embedded node, and if these forces exceed the fixed force of the node, it may cause the node to shift. The pressure in the holding stage is also an important factor affecting the displacement of the node, and a too high or uneven holding pressure will further aggravate the movement of the node. In addition, the uneven shrinkage caused by the different cooling rates in different regions during the cooling process will also cause slight displacement of the nodes.

**Figure 1:**
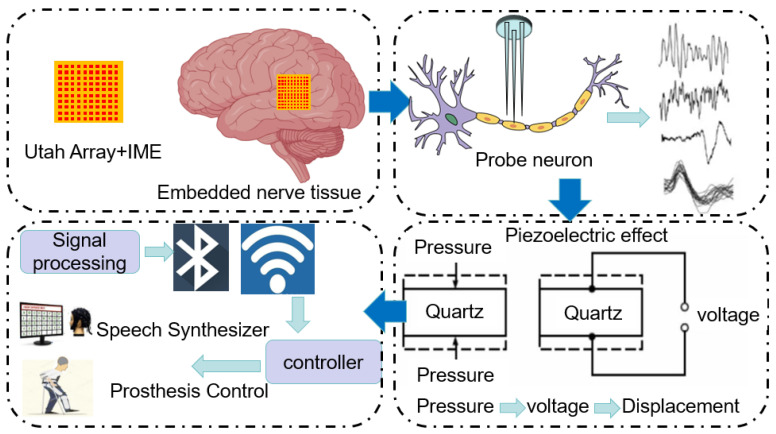
Implementation process of BCI.

The realization process of the brain–computer interface is shown in Figure 1. As technology advances and application requirements increase, the focus of research has gradually shifted to improving the performance, stability and user experience of the system. It is now more important to focus on the stability of the signal transmission as well as the stability of the received wave to ensure that the node displacement of the brain–computer interface remains within a small range. From the perspective of practical application, stable node displacement can effectively reduce the interference and loss in the signal transmission process and ensure the accuracy and stability of the brain–computer interface signal. In the application of the brain–computer interface, maintaining a small node displacement can help reduce signal noise, improve signal transmission efficiency, and ensure that the received brain waves and neural signals can accurately reflect the activity state of the brain.

Therefore, this paper focuses on the node displacement problem and explores methods and techniques to reduce node displacement by optimizing injection molding parameters. Although there are many other important research directions in the field of BCI, such as the improvement of signal-processing algorithms and the enhancement of hardware performance, the node displacement problem, as one of the core problems affecting the stability and accuracy of the system, is the basis for realizing an efficient and stable BCI system.

In the second section, a literature review of related research methods will be conducted to analyze the results and shortcomings of existing studies. The third section describes the materials and methods used in this paper, including the specific principles and formula derivation. The fourth section describes in detail the experimental program and the process of simulation. The fifth section describes and analyzes the result graphs obtained for the experiments and simulations. The last section summarizes the main conclusions of the research in this paper and discusses its significance and potential applications.

## 2. Literature Review

The focus of this paper is on the use of in-mold electronics (IME) to mold Utah arrays for the efficient manufacturing of brain–computer interfaces. IME technology allows for the simultaneous fabrication of electronic components and plastic housings during the molding process by embedding the conductive materials and functional components directly into the injection mold. This approach significantly improves product integration and performance while reducing manufacturing costs and complexity.

In this process, it is important to ensure precise node displacement in order to guarantee the quality of the lines in the film. Node displacement on the surface of the film is one of the key aspects of the entire signal transmission process, as the displacement of the line nodes directly affects the stability and accuracy of the signal transmission. Figure 1 shows the implementation of BCI, where when a piezoelectric sensor is used for signal transmission, small mechanical vibrations caused by neuronal activity result in node displacements on the surface of the film. These displacements are a product of the piezoelectric effect, which converts mechanical energy into electrical energy. Therefore, the accuracy and controllability of the initial node displacements directly determines the strength, stability and resolution of the signal. Therefore, the control of node displacement is crucial.

Brain–computer interface technology is currently in a rapid development stage and has made many impressive achievements. However, the technology still faces many challenges, one of which is that the node displacements of lines in microelectrode arrays can directly affect the quality of signal transmission and the stability of the device [15]. Nodal displacement of a line segment is the physical positional displacement of the line segment connection points due to material flow, cooling, and curing during the injection molding process. To ensure the minimum node displacement within the wire segment, the node displacement is optimized by adjusting the key parameters in the injection molding process.

Injection molding is a common manufacturing process used to heat thermoplastics to a molten state, then inject them into a mold and ultimately cool them for molding. During this process, the control of injection molding parameters has a significant impact on the quality and performance of the final product, especially for small electronic devices [16]. In the injection molding process, the precise control of process parameters is critical to product quality. Studies have shown that key parameters of injection molding, such as melt temperature, holding pressure, holding time, and cooling rate, have a direct impact on the physical properties and final quality of molded parts [17]. In recent years, many studies have focused on optimizing these parameters to reduce molding defects such as bubbles, warping, and shrink marks, and to improve the mechanical strength and surface finish of the products.

Especially in the high precision and complex geometries of molded parts, small parameter adjustments may also lead to significant quality differences [18]. Therefore, how to optimize the process through precise parameter control has become an important research direction to improve the stability of the injection molding process and product consistency [19]. These studies have further emphasized the importance of multiparameter optimization methods, proving that the residual stresses can be effectively reduced and the nodal displacements can be reduced through reasonable parameter combinations, thus significantly improving the overall performance of the molded parts [20].

There are many methods to optimize the injection molding parameters, such as applying a wavelet analysis to noise reduction and the feature extraction of the cavity pressure/holding pressure curves of injection molding machines, as well as identifying and optimizing the molding process parameters using a variety of recognizable performance evaluation methods [21]. Optimization techniques combining orthogonal tests, Kriging models and optimization algorithms can also be utilized. Kriging models are established using four types of correlation functions that affect the accuracy of the model, and the model with higher accuracy is selected [22]. The multi-objective optimization of the injection process can also be achieved by combining the Latin Hypercubic Sampling method with Constraint Generation Inverse Design Network (CGIDN) [23]. In optimizing the injection molding parameters, the NSGA-II algorithm was combined with a prediction model to obtain the Pareto optimal solution using the Kriging model [24]. A multi-objective robust optimization method using the Bayesian and Kriging models considering key metrics is used to study the deformation and temperature of plastic parts [25]. Among these optimization methods, the Kriging model is widely used in injection molding parameter optimization with its unique advantages. The Kriging model can provide very accurate prediction results. During the injection molding process, the accurate prediction of the effects of each parameter on the node displacement and other performance indexes helps to find the optimal parameter combination. Therefore, the Kriging model is chosen to optimize the injection molding parameters in this paper.

In order to find a more optimal combination of injection molding parameters, physically informative neural-network sparse regression control partial differential equations (PDEs) have good effectiveness and robustness [26]. A stochastic discretization method is used to optimize the PDE discretization for adaptive sparse mesh structures. The sparse regression of physically informative neural networks is used to control partial differential equations from sparse and noisy data in nonlinear spatiotemporal systems, proving the effectiveness and robustness of the method [27]. Deep-neural-network (DNN)-based methods can be used to enhance the scenarios of PDE systems using experimental data [28]. A combination of Kriging and partial differential equations was used to optimize the injection molding parameters to reduce node displacement. The effects of key factors on node displacement were analyzed using the Kriging model, which provided an important reference for optimizing the injection molding parameters. Meanwhile, the application of PDEs method further analyzes the heat transfer during the injection molding process in depth, providing a more comprehensive perspective for accurately determining the optimal injection molding parameters. The optimized parameters with minimum node displacements are finally determined, providing a reliable guarantee for stable signal transmission in applications such as brain–computer interfaces. The PDEs are capable of describing in detail the heat transfer and flow behaviors of the material during the injection molding process. The partial differential equation analysis can accurately simulate the flow state and solidification process of the material in the mold at different melting temperatures, thus evaluating the effect of temperature on the nodal displacement and the quality of the finished product. Therefore, the partial differential equation analysis can be combined with the Kriging model to further improve the efficiency and accuracy of the optimization process. The Kriging model provides preliminary parameter optimization schemes, while the PDEs analysis verifies and optimizes these schemes in detail to ensure that the final parameter combinations achieve the best performance in practical applications.

In this paper, the injection molding process needs to be finely optimized in order to ensure the nodal displacement of the film surface. This may involve the choice of material, the design of the film structure, and the size and shape of the device. By combining the optimization of the injection molding parameters, the desired node displacement characteristics can be achieved, thus improving the performance and effectiveness of the brain–computer interface.

**Figure 2:**
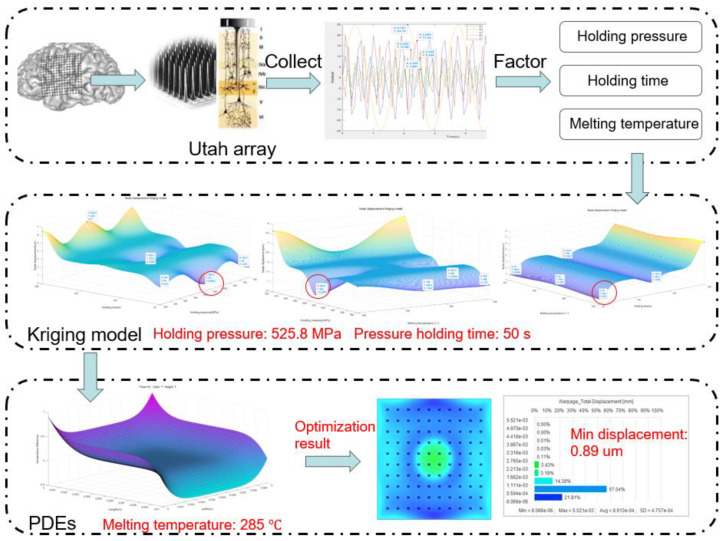
Optimized brain–computer interface framework.

As shown in Figure 2, it is the overall framework diagram of the optimized brain–computer interface in this paper. The injection molding method of in-mold electronic decoration is an advanced technique to embed the electronics directly into the brain tissue to provide more stable and accurate signal acquisition, thus overcoming the instability and interference problems of traditional external electrodes and providing a more accurate input source for the BCI system. Utah arrays, as electrode arrays for brain–computer interfaces, have excellent spatial resolution and signal capture capability, and can record the activities of a large number of neurons simultaneously, thus realizing a more comprehensive understanding of brain activities and providing more diverse and accurate input signals for brain–computer interface systems.

The Kriging prediction model is combined with PDEs to optimize the injection parameters of the brain–computer interface to further improve the performance of the brain–computer interface system. The PDEs model models and simulates the physical processes of the brain–computer interface and helps to select the optimal injection parameters for optimal electrode positions and layouts. The PDEs approach takes into account the effect of the injection parameters on the node displacements of the brain–computer interface, which allows for a more accurate description of the physical phenomena. The effect of injection molding parameters on brain–computer interface node displacements is investigated. The Kriging prediction model, as a powerful statistical tool, can accurately estimate the effect of injection molding parameters on performance based on the spatial distribution of available data points. Finally, the brain–computer interface node displacements before and after optimization are analyzed by combining Kriging and PDEs to verify their effects on signal transmission accuracy. This approach provides us with a comprehensive analytical tool to ensure that the performance of the BCI system is fully evaluated.

## 3. Materials and Methods

### 3.1. Materials

Liquid silicone rubber (LSR) is an advanced elastomer with excellent flexibility, biocompatibility and high temperature resistance, suitable for the manufacture of flexible electrode arrays. LSR materials maintain their mechanical properties and elasticity at high and low temperatures and in various chemical environments, demonstrating excellent aging resistance. As a result, LSR can maintain its excellent mechanical properties and elasticity over a long period of use [29]. In addition, LSR has good mechanical adaptability when implanted into brain tissue, which can reduce the damage to brain tissue during implantation and maintain a relatively stable implantation position [30].

Polyethylene terephthalate (PET) is a hard and transparent polymer material with excellent chemical resistance and stability [31]. PET is commonly used to make rigid electrode substrates or wires that provide support and stability for the electrode array, thus ensuring the good positioning of the electrode in the brain tissue.

**Figure 3:**
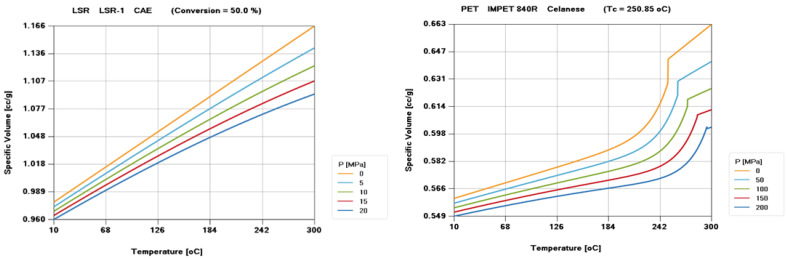
PVT diagram of LSR and PET materials.

The combination of LSR and PET gives full play to the advantages of each in the brain–computer interface. The flexible LSR electrode array ADAPTS to the shape of the brain tissue and reduces the damage to the brain tissue after implantation, while PET provides a stable support structure to ensure that the electrode maintains good position and stability after implantation. By combining the injection molding process of PET with the in-mold decoration technology of LSR, the precision manufacturing and external decoration of Utah array microelectrodes can be achieved, providing an innovative solution for the performance and reliability of brain–computer interfaces. As shown in Figure 3, the PVT performance and material model of PET and LSR materials selected in this paper have been fully demonstrated.

### 3.2. Methods

#### 3.2.1. Kriging Prediction

Approximate models are constructed from actual analyzed data to cope with the huge computational volume and complexity of the numerical simulation models [32]. In this context, the Kriging proxy model becomes a common tool. The Kriging proxy model is a generalized regression model based on the stochastic process. Its core idea is to predict unknown sample points by known sample points and to reflect spatial changes by changes in variance. This method can effectively reduce the workload of simulation calculation and improve the solution efficiency [33]. The Kriging model has the characteristics of better local estimation, especially when dealing with nonlinear problems, and its accuracy is relatively high, so it is widely used in engineering application analysis.

Input variables and response values of *m* observation points are known, being denoting as $S_{m}=$ [*x*1, *x*2, …, *xm*] and Y = ${\left[y1,y2,\ldots ,ym\right]}^{T}$, where $x_{i}\in R^{n}$, $y_{i}\in R$, *i* = 1, 2, …, *m*. Now, it is necessary to interpolate and analyze the response value *y* of unknown *x*. Now, the relation expression between input variables and response value in the Kriging model is written as Equation (1).

(1) $$y\left(x\right)=F\left(\beta ,x\right)+z\left(x\right)=f^{T}\left(x\right)\beta +z(x)$$

*m* and *n* are the dimensions of design variable *x*. *F*(*β*,*x*) in Equation (1) is a linear regression model of data, and the number of regression vectors reflects the change in mean value in the fitting process of the model. The linear regression model *p* = 1, $f_{1}(x)$ = 1 is used in practical engineering application. *z*(*x*) is a stochastic process model established by data observation and the quantification of data correlation, whose mean value is θ and variance is $\sigma^{2}$. The covariance between sampling points is shown in Equation (2).

(2) $$\left\{\begin{array}{c} E\left\{z\left(x\right)\right\}=0\\ Var\left[z\left(x\right)\right]=\sigma^{2}\\ Cov\left[z\left(x_{i}\right),z\left(x_{j}\right)\right]=\sigma^{2}[R(\theta ,x_{i},x_{j})] \end{array}\right.$$

SCF R(θ,w,x) mathematical expression is shown in Equation (3), in which *θ* is a key parameter of the Gaussian correlation function. The correlation between sampling points can be adjusted adaptively by optimizing *θ*.

(3) $$R\left(\theta ,w,x\right)=\prod_{i=1}^{n}R_{i}(\theta_{i},w_{i}-x_{i})$$

Spatial correlation functions play an important role in Kriging models by controlling the smoothness of the model, describing the interrelationships between nearby points, and quantifying the correlation between observations. This allows the Kriging model to treat any response value as a random variable obeying a normal distribution without being constrained by a specific form, showing a high degree of flexibility.

The standard Kriging model usually uses seven common spatial correlation functions, including the exponential model, the exponential Gaussian model, the Gaussian model, the linear model, the spherical model, the cubic model, and the spline function model. Among them, the Gaussian model is a commonly used spatial correlation function, with the expression shown in Equation (4). This model can provide a relatively smooth and infinitely differentiable surface, so it is widely used in practical analytical applications.

The smoothness and fine-tunability of Gaussian models make them ideal for many practical applications, as they can effectively capture trends in space while providing feasible computational efficiency in real-world applications. This flexibility and tunability makes the Kriging model a powerful tool for processing spatial data and performing predictive analysis.

(4) $$R_{i}\left(\theta_{i},d_{i}\right)=\exp(-\theta_{i}{\left|d_{i}\right|}^{2})$$

The function related to the type *R* on the distance between two points with $\left|w_{i}-x_{i}\right|$ and change, by using maximum likelihood estimation theory and unconstrained optimization methods, can maximize Equation (5). The optimal value of *θ* can be calculated as follows:

(5) $$-\left(mln\sigma^{2}+ln\left|R\right|\right)/2$$

In practice, pursuing the optimal *θ* value does not guarantee the best approximation curve, but the approximation results usually improve significantly when the *θ* value is close to the optimal solution. In our study, we used MATLAB(2020a) software for the construction and optimization-seeking computation of the Kriging agent model. MATLAB has built-in tools for experimental design, model building, and computation, which allow us to adjust the parameters more flexibly.

By building a predictive model based on the spatial distribution of known data points, Kriging provides accurate estimates for node displacements. This approach takes into account not only the spatial relationships between nodes, but also the uncertainty factor, allowing us to more accurately adjust the injection parameters to achieve the goal of minimizing node displacements.

#### 3.2.2. Regression Partial Differential Equations (PDEs)

The regression partial differential equations (PDEs) [34] method is a mathematical method based on the combination of partial differential equations and regression models. It is based on the principle of using partial differential equations to describe and model the dynamic behavior of a system or process, and then estimating the unknown parameters in these partial differential equations based on actual data through regression analysis to optimize the performance of the system or predict the future behavior.

In using recognizable partial differential equations applied to the Utah array, the main steps are as follows:

1. Heat Transfer Modeling: The Utah array injection molding process is modeled using the heat transfer equation, incorporating material thermal properties like conductivity and specific heat capacity. Melting temperature is a key parameter.
2. Numerical Solution: the heat transfer model is numerically solved to generate temperature distribution and heat transfer data under varying melt temperatures.
3. Nodal Displacement Analysis: analyze nodal displacement trends based on numerical results to identify the optimal melt temperature that minimizes displacement.
4. Optimization: use numerical optimization algorithms to find the ideal melt temperature that minimizes nodal displacement, considering process constraints.
5. Validation and Adjustment: implement the optimized melt temperature in the injection molding process, validate through experiments, and adjust the model to ensure alignment with real-world results.

We consider a multi-dimensional spatiotemporal system whose governing equations can be described by a set of nonlinear, coupled, parameterized PDEs in the general [35] form given by Equation (6):

(6) $$u_{t}+F\left[u,u^{2},\cdots \nabla_{x}^{2}u,\nabla_{x}u\cdot u,\cdots ;\lambda \right]=p$$

where $u=u(x,t)\in R^{1\times n\;}$is the multi-dimensional latent solution (dimension = n) w h i l e ut is the first-order time derivative term; t ∈ [0, T] denotes time and x ∈ Ω specifies the space; $F\left[\cdot \right]$ is a complex nonlinear functional of u and its spatial derivatives, parameterized by λ; ∇ is the gradient operator with respect to x; p=p(x, t) is the source term (note that, in many common cases, p=0 represents no source input to the system). The PDEs are also subjected to initial and boundary conditions (I/BCs), if known, denoted by $L\left[x\in \;\Omega ,t=0;u,u_{t}\right]=0\;and\;B\left[x\in \partial \Omega ,u,\nabla_{x}u\right]=0$.

For systems that obey Newton’s second law of motion (e.g., $u_{tt}$ in wave equations), the governing PDEs can be written in a state-space form of Equation (6) by defining $v=\left\{u_{tt}\right\}$ as the solution variable. Our objective is to find the closed form of $F\left[\cdot \right]$ from the available spatiotemporal measurements which are assumed to be incomplete, scarce and noisy, commonly seen in real-world applications (e.g., when data capture is very costly or the data itself is sparse in nature). We assume that the physical law is governed by only a few important terms which can be selected from a large-space library of candidate functions, were sparse regression.

Constant, polynomial, and trigonometric terms with respect to each spatial dimension, 6, 7, assembled in a row vector given by $\phi =\left\{1,u,u^{2},\cdots u_{x},u_{y},\cdots u^{3}⨀u_{xy},\cdots ,sin(u),\cdots \right\}$, where ⊙ represents the element-wise Hadamard product; s denotes the total number of candidate terms in the library; the subscripts in the context of {x, y, z} depict the derivatives;$\;\Lambda \;\in R^{s\times n\;}$ is the sparse coefficient matrix (only the active candidate terms in ϕ have nonzero values), e.g., $\Lambda =[\lambda^{u}\lambda^{v}\lambda^{w}]\in R^{s\times 3\;}$ for u={u, v, w}. If there is an unknown source input, the candidate functions for p can also be incorporated into ϕ for discovery. Thus, the discovery problem can then be stated as: given the spatiotemporal measurement data${\;D}_{u}$ find sparse Λ such that Equation (7) holds.

(7) $$u_{t}=\emptyset \Lambda$$

We present a new PINN-SR paradigm to simultaneously model the system response and identify the parsimonious closed form of the governing PDE(s). The innovative algorithm architecture of this method, where datasets sampled from two different I/BC scenarios are considered: (1) one dataset from a single I/BC and (2) *r ≥* 2 independent datasets from multiple I/ BCs. For the case of single dataset, we interpret the latent solution u by a DNN (denoted by N), namely, $u_{i}^{\theta }=u(x,\;t;\;\theta )$, where *θ* represents the DNN trainable parameters including weights and biases. When multiple independent datasets are available, a “root-branch” DNN is designed to approximate the latent solutions $u_{i}\;(i=1,\;\ldots ,\;r)$ corresponding to different I/BCs, viz., $u_{i}^{\theta }=u(x,t;\theta^{\left(0\right)},\theta^{\left(i\right)})$, where $\theta^{\left(0\right)}$ and $\theta^{\left(i\right)}$ denote the trainable parameters of the root layers $N^{\left(0\right)}$ and the branch layers $N^{\left(i\right)}$, respectively. Noteworthy, the I/BCs are unnecessarily either known a priori or measured, since the measurement data already reflects the specific I/BC (e.g., there exists a one-to-one mapping between the I/BC and the PDE solution). The DNN essentially plays a role as a nonlinear functional to approximate the latent solution with the data loss function $L_{d}(\theta ,D_{u})$. With automatic differentiation where derivatives on u are evaluated at machine precision, the library of candidate functions $\phi^{\theta }$ can be computed from the DNN. For the case of multiple independent datasets, the libraries $\phi^{\left(i\right)}$ resulted from the branch nets are concatenated to build $\phi^{\theta }$ for constructing the unified governing PDEs. Thus, the sparse representation of the reconstructed PDE(s) can be written in a residual form, namely, $R^{\theta }=u_{t}^{\theta }-\phi^{\theta }⋀\to 0,\;where\;R^{\theta }\in R^{1\times n}$ denotes the PDE residuals. The basic concept is to adapt both the DNN trainable parameters θ and the PDE coefficients Λ, such that the neural network can fit the measurement data while satisfying the constraints defined by the underlying PDEs. The PDE residuals will be evaluated on a large number of collocation point $D_{c}={\left\{x_{i},t_{i}\right\}}_{i=1}^{N}$, randomly sampled in the spatiotemporal space, leading to the residual physics loss function $L_{p}(\theta ,\Lambda ,D_{c})$. When multiple I/BCs are considered, the measurement data and the collocation points will be stacked when calculating the data loss and the physics loss (based on a unified physics residual formulation $R^{\theta }\to 0$.

The total loss function for training the overall PINN-SR network is thus composed of the data loss $L_{d}$, the residual physics loss$\;L_{p}$ and a regularization term, expressed as Equation (8):

(8) $$L_{p}(\theta ,\Lambda ,{D_{u},D}_{c})=L_{d}(\theta ,D_{u})+{\alpha L}_{p}(\theta ,\Lambda ,D_{c})+\beta {\left\|\Lambda \right\|}_{0}$$

where α is the relative weighting of the residual physics loss function; β is the regularization parameter; and ${∥\;\cdot \;∥}_{0}$ represents the $l_{0}$ norm. Optimizing the total loss function can produce a DNN that can not only predict the data-driven full-field system response, but also uncover the parsimonious closed-form PDE(s), i.e., $\left\{\theta^{*},\;\Lambda^{*}\}:=arg\;{min}_{\left\{\theta ,\Lambda \right\}}[L(\theta ,⋀,{D_{u},D}_{c})\right]$, where $\left\{\theta^{*},\;\Lambda^{*}\right\}$ denote the optimal set of parameters. It is worth noting that the total loss function has an implicit complex form, and thus, directly solving the optimization problem is highly intractable since the $l_{0}$ regularization makes this problem np-hard. To address this challenge, we present an alternating direction optimization (ADO) algorithm that divides the overall optimization problem into a set of tractable subproblems to sequentially optimize the trainable parameters. The pre-training of PINN-SR is conducted before running the ADO algorithm for discovery, by simply replacing ${∥\Lambda ∥}_{0}$ in Equation (8) with ${∥\Lambda ∥}_{1}$ where brute-force gradient-based optimization for both θ and Λ becomes applicable.

The $l_{1}$-regularized pre-training can accelerate the convergence of ADO by providing an admissible “initial guess”.

**Figure 4:**
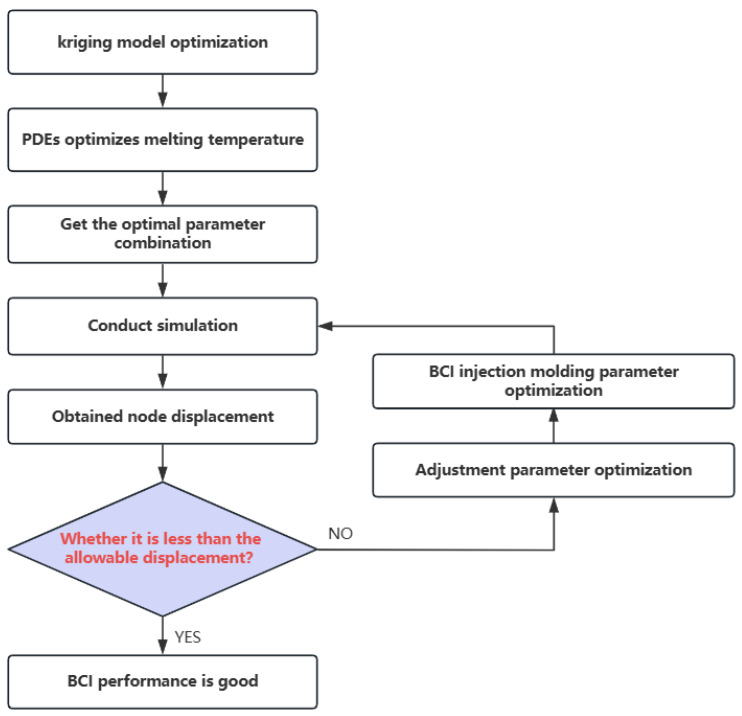
BCI optimization and demonstration process.

In order to verify whether the optimized brain–computer interface can achieve accurate signal transmission, we use the relationship between line diameter and current and voltage to derive the maximum allowable node displacement, so as to verify whether the parameter combination is feasible. The maximum allowable value of node displacement is calculated in the following section. As shown in the flow diagram in Figure 4, this flow is a demonstration of the entire optimization process and the proposed signal transmission accuracy to ensure that the BCI can transmit EEG signals stably and accurately in practical applications.

#### 3.2.3. Relationship between Wire Diameter and Current

In Utah arrays or other implantable electrode arrays, ensuring the stability of the leads is critical for reliable recording and transmission of EEG signals. Changes in the lead wires may arise from a variety of factors, including tissue response after implantation, mechanical displacement, or other physiological changes. These changes may lead to the deterioration of the contact between the electrode and the neuron, thus affecting the signal quality.

Throughout the implementation of a brain–computer interface, brain signals are captured onto an array of implantable electrodes and connecting wires transmit these signals to a specially designed ECOG amplifier. Offset or length variations in the line can affect signal transmission by introducing propagation delays, phase changes and possible signal distortion. This is because changes in line length result in changes in the propagation speed, phase, and frequency response of the signal, which can lead to impedance mismatches, signal reflections, and time differences at the receiving end. In high-frequency applications or applications that require high signal quality, engineers need to take steps, such as selecting the proper type of transmission line, adding compensation circuits, or using special signal processing techniques, to ensure accurate signal transmission.

For a copper wire, the resistivity ρ is about $1.68\times 10^{-8\;}\Omega \cdot m$. In this case, for every 0.01 mm change in line diameter, the change in current can be estimated using Equation (9):

(9) $$\frac{dI}{dd}=-\frac{V}{2\rho }\cdot \frac{1}{{\left(\frac{d}{2}\right)}^{2}}$$

Equation (10) indicates that an increase in line diameter will result in an increase in current, while a decrease in line diameter will result in a decrease in current. The exact magnitude of the change will depend on the initial current value and the exact amount of change in the line diameter. By using the formula, we realize that for every 0.01 mm change in line diameter, the current will change by 0.355 A, and next we would like to use this change to figure out the range of the order in which the node displacements will change in a thin film circuit with an initial diameter of 0.1 mm.

(10) $$∆I=\frac{V}{2\rho }\cdot \frac{1}{\left(\frac{d+∆d}{2}\right)}$$

The calculated range of allowable wire diameter variation is 0.017 mm, which means that within this range, the current variation will keep the current stabilized and the signal transmission through is relatively stable.

Therefore, it is important to study changes in node displacement to understand the stability and accuracy of electrical signal recordings. Such studies may include improvements in wire fixation methods, post-implantation biological tissue response, the optimization of wire materials, and the investigation of other engineering and biological factors related to electrode stability.

## 4. Case Study

In order to make brain–computer interfaces (BCIs) smaller and lighter for implantation, we have adopted in-mold electronics (IME) technology and injection molding using liquid silicone rubber (LSR) materials. The application of IME technology not only enables complex electronic components to be embedded directly into thin films, but also ensures the reliability and durability of the electronic components through a high-precision injection molding process. During the injection molding process, Utah arrays are precisely embedded into the flexible film, resulting in BCIs that are small in size, light in weight, and stable in performance.

**Figure 5:**
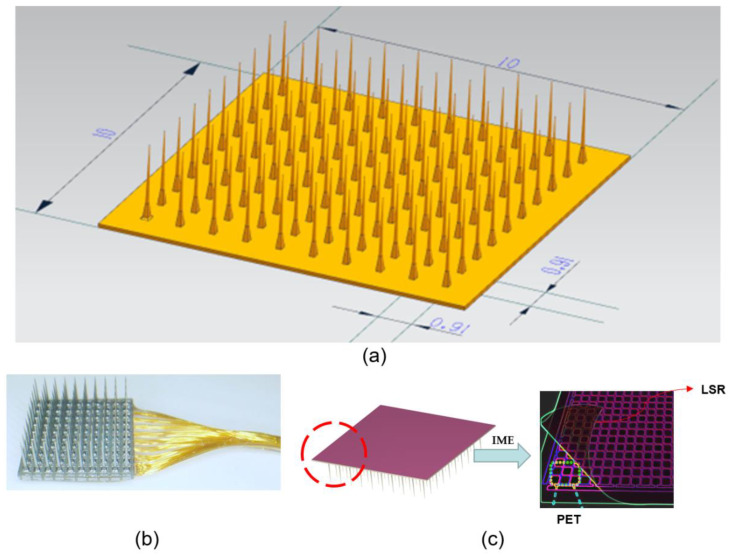
Design of Utah array model. (**a**) The three-dimensional model of Utah array and its size parameters are shown. (**b**) the actual model of Utah array is shown. (**c**) The Utah array is shown under the principle of IME technology.

As shown in Figure 5a, we designed the 3D model of the Utah array as a 10 × 10 mm square electrode plate with 100 tiny electrodes. For the Utah array, as shown in Figure 5b, there is usually one connection line for each channel. This means that for 100 channels, there are typically 100 connection lines [5]. These connecting lines are responsible for transmitting the signals from each channel to the appropriate multiplexer or demultiplexer and then to the external system.

When designing such a system, it is important to ensure that each connecting line transmits data independently without interfering or affecting the other lines. This may involve designing circuit boards, connectors, cables, or other equipment necessary to ensure that each connecting line can transmit data consistently. It is also necessary to ensure the reliability and stability of the entire system to avoid possible data loss or transmission errors.

Based on the literature research, this paper establishes 100 interface channels by running the Utah array of probes through the entire electrode plate [10], providing wider coverage and higher acquisition accuracy for the signal acquisition and transmission of the brain–computer interface. The 100 channels were divided into two groups, yellow and green, with 50 channels in each group to reduce interference and cross-talk between signals and improve the accuracy and stability of signal transmission [36]. In addition, after covering a layer of biocompatible LSR film, the array and connecting lines can be effectively protected from the external environment, improving the stability and durability of the system and extending the service life of the device. Therefore, the design method of Figure 5c is chosen in this paper.

**Figure 6:**
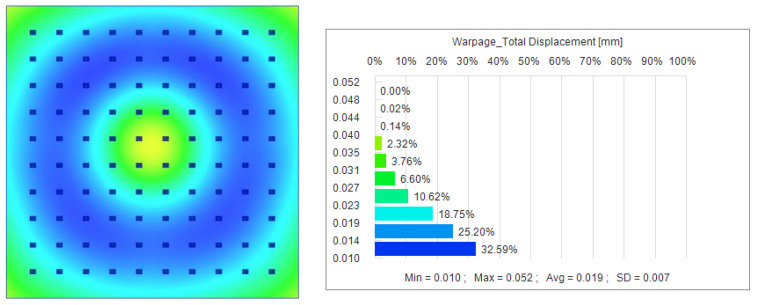
Node displacements obtained from initial conditions.

In Utah arrays, it is critical to ensure that the node displacements of the lines are not offset. Offsets in node displacement can lead to data inaccuracies, instability between array elements, and an increased risk of surgery. Maintaining the stability of the nodes of the Utah array line is critical to ensure its performance and reliability. We focus on optimizing node displacement by optimizing injection parameters.[37]. Melt temperature affects the fluidity and melt state of the plastic, injection speed affects the filling of the plastic in the mold cavity, and holding time and holding pressure determine the shrinkage and stability of the plastic during the cooling and curing stages. We used the initial data for the experimental study with a holding pressure of 750 MPa, a holding time of 175 s, and a melting temperature of 285 °C for the simulation in Moldex3D version 2023 software, and obtained the results shown in Figure 6. The maximum node displacement is 0.052 mm, the minimum is 0.010 mm, and the average node displacement is 0.19 mm, a value that may have a serious negative impact on the brain–computer interface’s functionality.

To further improve the performance and stability of the brain–computer interface, we need to optimize the injection parameters. This will involve more experimentation and data collection to determine the optimal combination of injection molding parameters that significantly reduces node displacement and improves the stability and performance of the interface. By optimizing key parameters such as holding pressure, holding time, melting temperature, etc., we hope to reduce the node displacement to a negligible level, thus enhancing the overall functionality of the brain–computer interface.

In this paper, we propose an injection molding parameter optimization method by combining the Kriging prediction model with partial differential equations (PDEs.) Kriging is a statistically based method that can provide a reliable basis for parameter optimization in the injection molding process by analyzing and predicting known data points. Partial differential equations are used to describe the complex physical phenomena of material flow, heat transfer and curing during the injection molding process. By combining these two methods, we can more comprehensively understand and control the effects of injection molding parameters on the molding process.

We first use the Kriging prediction model to perform a preliminary optimization of key parameters such as holding pressure, holding time and melt temperature during the injection molding process. Based on the prediction results of this model, the optimized parameter values were substituted into partial differential equations for simulation to evaluate the effects of different parameter combinations on nodal displacement. The simulation was carried out using Moldex3D version 2023 software. Through several iterations of optimization and simulation, we finally determined the optimal combination of injection parameters to significantly reduce node displacement and improve the stability and performance of the brain–computer interface.

## 5. Results and Discussion

In conducting the Kriging model analysis, we first adopted the orthogonal experimental design method to ensure the comprehensiveness and efficiency of the experimental design. Specifically, the holding pressure was set at 300–750 MPa, the holding time was set at 50–200 s, and the melting temperature was set at 285–300 °C. In order to simulate the effects of different parameter combinations on the nodal displacements, we performed simulations in Moldex3D.

In the orthogonal experimental design, we chose three levels of holding pressure, holding time and melting temperature, corresponding to low, medium and high values within the set range. Twenty sets of data were selected, as shown in Table 1. The displacements here were obtained by simulating different combinations of parameters and by setting the nodes in the simulation software. These data provide a solid foundation for the subsequent Kriging prediction model.

We set up several key nodes in the simulation model, which are located on the lines in the film designed to receive EEG signals. During the simulation process, the software will record the position change in each node to obtain the node displacement. Through multiple simulations and parameter adjustments, we can obtain the node displacement data under different combinations of injection molding parameters.

**Figure 7:**
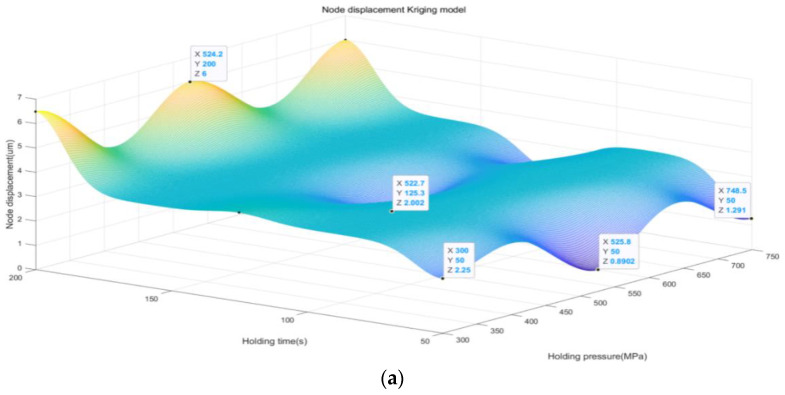

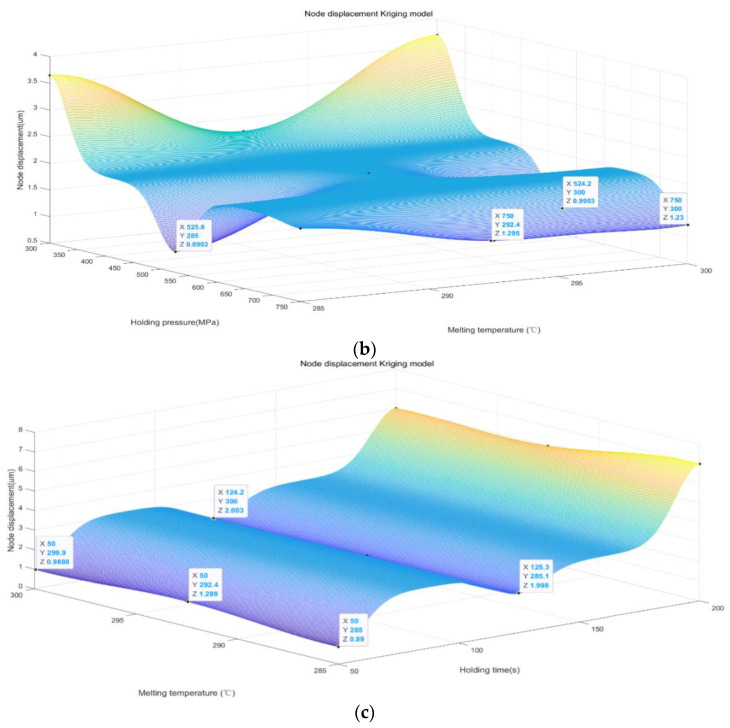
Kriging prediction of nodal displacement by three factors: (**a**) the effect of holding pressure vs. holding time; (**b**) the effect of holding pressure vs. melt temperature; (**c**) the effect of holding time vs. melt temperature.

The Kriging model generates a predictive model of node displacement by interpolating known data points. Based on this model, we can predict the node displacements under different combinations of injection molding parameters, thus identifying the optimal possible parameter combinations. Therefore, with the above 20 sets of data, we use the Kriging model to analyze and predict the simulation data and obtain the result graph, as shown in Figure 7.

First, we observed that an increase in the holding pressure in the range of 300–750 MPa had a significant effect on the node displacement of the BCI. As the holding pressure increases, we observe a relative decrease in node displacement, which suggests that increasing the holding pressure within a certain range can effectively improve the stability and accuracy of the BCI. This result highlights the important influence of holding pressure on the performance of BCI and suggests that the optimization of holding pressure should be fully considered when designing BCI.

Second, the decrease in the holding pressure time in the range of 50–200 s also had a significant effect on the node displacement. We found that the node displacement relatively decreases with the decrease in holding pressure time, indicating that a shorter holding pressure time can help to reduce the displacement problem of the BCI and thus improve its stability and performance. This finding emphasizes the importance of precisely controlling the holding pressure time during the preparation of BCI to ensure that the BCIs can exhibit stable and reliable properties in practical applications.

Finally, although we note that the melting temperature in the range of 285–300 °C has less effect on the node displacement, it still affects the performance of the BCI to some extent. This suggests that the appropriate choice of melting temperature is still one of the crucial factors in the preparation process of BCI. The comprehensive consideration of the effects of holding pressure, holding time, and melting temperature on node displacement can further optimize the design of BCI and improve their stability and performance, so as to better meet the needs of practical applications.

Through our prediction model, we find that the minimum node displacement is only 0.89 µm, which indicates that we are able to achieve a very high level of node displacement control under a certain holding pressure, holding time, and melting temperature. Specifically, we found that the performance of the BCI was optimized at a holding pressure of 525 MPa, a holding time of 50 s, and a melt temperature of 285 °C.

**Figure 8:**
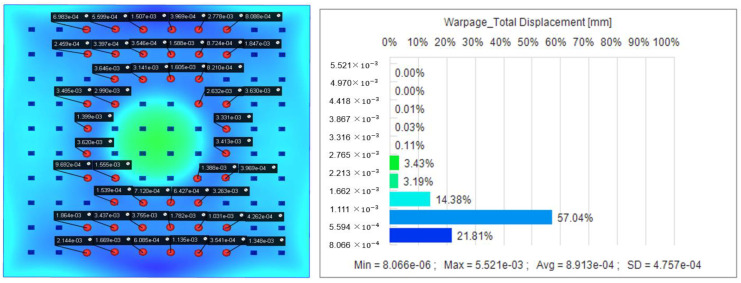
Displacement distribution of nodes after optimization.

The resultant plots of node displacements are obtained by simulation analysis using the optimal parameter combinations obtained from the Kriging prediction model. As shown in Figure 8, the maximum displacement was reduced from 0.019 mm to only 0.89 µm with an optimization rate of 95.32%. This shows that the Kriging method has a significant role in improving the nodal displacement.

In our observations of the optimized Utah array, we found that the probe node displacements at the positions shown in Figure 8 ranged from 0.008 µm to 0.39 µm. Based on this observation, we decided that the signals obtained from the probes at these locations are usually selected for analysis during signal acquisition. This strategy is based on a thorough consideration of the distribution of node displacements and helps ensure that the signal data we obtain is more accurate and reliable. By focusing on these four locations, we are able to capture the neural signal information transmitted by the BCI more accurately and avoid the interference caused by the displacement of probes in other regions on the signal acquisition and analysis process.

Specifically, melt temperature plays a critical role in the injection molding process, directly affecting material flow, filling properties, and the mechanical and electrical characteristics of the final product. Within the constraints of the material selected, even small temperature variations may significantly affect nodal displacement and product quality, although the range of melt temperatures is small. Therefore, with PDEs analysis, it is possible to accurately simulate the behavior of the material at different melting temperatures and find the temperature value that is most conducive to reducing nodal displacement. In the PDEs analysis, we constructed a mathematical model describing the heat transfer and material flow during the injection molding process. The model takes into account the effect of melt temperature on material viscosity, thermal conductivity and density, which enables us to simulate the material filling and curing process at different temperatures.

In the analysis of PDEs, we focused on the variation of the melting temperature in the range of 285–300 °C. The results are summarized in the following sections. First, we constructed a mathematical model which takes into account the effect of the melting temperature on the viscosity, thermal conductivity and density of the material. By setting different melt temperatures, we simulated the flow and solidification process of the material in the mold.

In this study, in order to further optimize the nodal displacements, we first focused on the variation of the melting temperature in the range of 285–300 °C by Kriging analysis, and came up with the optimal value of 285 °C for the melting temperature. Based on this result, we performed a detailed analysis of the melting temperature of 285 °C using the partial differential equations (PDEs) method.

**Figure 9:**
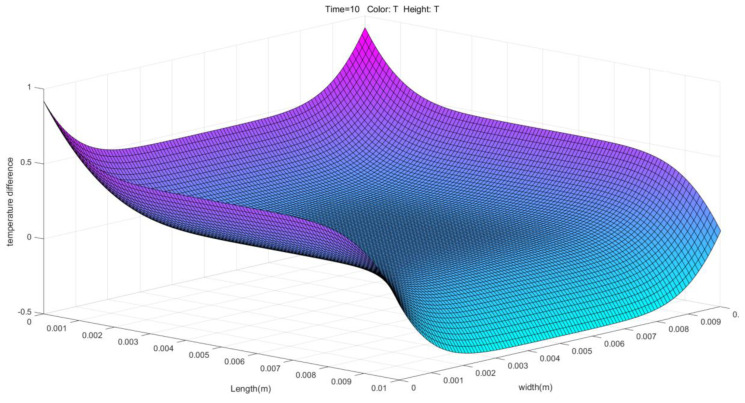
Heat transfer two-direction displacement for Utah array molding.

Through the simulation and analysis of the PDEs method, we obtained the three-dimensional temperature distribution graph shown in Figure 9 in MATLAB. This figure shows the temperature distribution of the material in the mold at the melting temperature of 285 °C. Figure 9 shows that the temperature difference is small and the overall temperature change is relatively smooth. The small temperature gradient means that the temperature changes at various points within the material are not significant, which helps to reduce the uneven material shrinkage due to temperature differences and further reduces the nodal displacement. The temperature distribution is more stable over a length and width range of 0.01 mm. This indicates that at this temperature, the flow and filling process of the material is more stable, and there is no obvious overheating or uneven cooling phenomenon.

Based on the above analysis, we confirm that a melt temperature of 285 °C provides optimal temperature conditions during the injection molding process. This temperature not only ensures good fluidity and the uniform temperature distribution of the material, but also effectively reduces nodal displacement and improves the stability and performance of the brain–computer interface.

Combining the Kriging prediction model and partial differential equations (PDEs) method, we optimized the key parameters in the injection molding process and finally determined the optimal combination of injection molding parameters: a holding pressure of 525 MPa, a holding time of 50 s, a melting temperature of 285 °C, and a nodal displacement of 0.89 µm. This combination greatly reduced nodal displacement and improved stability and performance.

From the results, the melting temperature of 285 °C ensured uniform flow and the stable filling of the material, which was essential to reduce the nodal displacement caused by the temperature difference. The smoothness of the temperature distribution reduces the shrinkage inhomogeneity of the material, which is essential to maintain the precise position of the electronic components in the Utah array, thus improving the accuracy and reliability of signal transmission.

The combination of the Kriging model and PDEs methodology provides an effective tool for injection molding parameter optimization. The Kriging model provides a theoretical basis for parameter optimization by analyzing and predicting known data points, while the PDEs method describes in detail the physical behavior of the material during the injection molding process, such as flow and cure. The combination of the two allows us to accurately assess the effects of different parameter combinations on nodal displacement in simulations, and thus find the optimal injection molding conditions.

The research in this paper was conducted based on simulation models and no actual objects were developed. All optimization results and conclusions are theoretical, and future work will include the fabrication of actual samples and experimental validation to further verify the effectiveness of the simulation results for practical applications. Although these methods performed well in our study, their adaptability and generalization ability under different materials and process conditions still need to be further verified. In actual production, various uncertainties and process variations may be encountered, which may affect the accuracy of model predictions. Therefore, the model needs to be further validated and adapted in the future to meet the challenges in actual production.

Errors may exist between experimental and simulated data, and thus these errors need to be analyzed and corrected to ensure the accuracy and reliability of the model. Through an in-depth analysis of the consistency problems of experimental and simulated data, as well as the technical bottlenecks in practical applications, we can more clearly recognize the limitations of the existing methods and take improvement measures in future research to further enhance the optimization effect and application value of the injection molding process.

## 6. Conclusions

In this paper, based on summarizing the previous research work, we introduced the domestic and international development status and trend of embedded brain–computer interfaces using Utah arrays, and carried out an in-depth analysis and optimization of the brain–computer interface injection molding process. We have successfully improved the relevant process parameters through a variety of advanced technological means to address the stability and signal transmission challenges of the Utah array, and have come up with the following main conclusions:

1. Innovations in Material–Process Combination: By combining Polymer Enclosure Technology (PET) with liquid silicone rubber (LSR) and using Internal Modeling for Real-Time Monitoring (IME) of the electronics, we have successfully prepared a high-performance Utah array. This combination improves the stability of the BCI and the reliability of signal transmission, and lays a solid foundation for more complex applications in the future.
2. The Kriging model to optimize injection parameters: Using the Kriging prediction model, we optimized the process parameters, such as holding pressure and holding time. The results show that the holding pressure is the main factor affecting the node displacement, and its optimized node displacement minimum value is 0.89 µm, which is 95.32% higher than the initial state. This optimization significantly improves the transfer accuracy and stability of BCI.
3. Multi-parameter optimization study of the system: In this study, not only the influence of a single parameter is considered, but also a systematic optimization combination of several key parameters (e.g., holding pressure, holding time, and melting temperature) is carried out. Through the comprehensive optimization of multiple parameters, we successfully achieved the best injection molding results and verified that the optimized node displacement complied with the allowable range (0.0017 mm) derived from Ohm’s law.
4. Theoretical analysis and validation: By combining the technique of identifiable partial differential equations (PDEs), we conducted an in-depth study on the heat transfer during the injection molding process. The optimized results are highly compatible with the theoretically predicted data, further confirming the effectiveness of the optimization measures and the improvement of BCI performance.

These findings not only directly respond to the goal and hypothesis at the beginning of the research, but also provide important theoretical and technical references for the future development and application of brain–computer interface technology. In the future, we will continue to explore more advanced technical means to further improve the performance and stability of brain–computer interfaces.

## Figures and Tables

**Table 1:** The influence of three factors on node displacement after optimization.

Run	A: Holding Pressure	B: Holding Time	C: Melting Temperature	Node Displacement
	MPa	s	°C	μm
1	525	50	285	0.89
2	750	50	292.5	1.29
3	750	125	285	2.00
4	525	200	300	6.00
5	525	125	292.5	2.00
6	300	50	292.5	2.25
7	525	125	292.5	2.00
8	525	125	292.5	2.00
9	300	125	285	3.65
10	525	125	292.5	2.00
11	750	125	300	2.00
12	525	200	285	7.00
13	300	200	292.5	6.49
14	525	125	292.5	2.00
15	750	200	292.5	6.00
16	525	50	300	0.99
17	300	125	300	3.70

## Data Availability

The authors declare that the data supporting the results of this study are available in the paper. If any raw data files in other formats are required, they can be obtained from the corresponding author upon reasonable request.
